# Conducting the National Health and Morbidity Survey 2023 in Malaysia with focus on methodology and main findings on non-communicable diseases

**DOI:** 10.1038/s41598-025-08311-9

**Published:** 2025-07-15

**Authors:** Halizah Mat Rifin, Muhammad Fadhli Mohd Yusoff, Hamizatul Akmal Abd Hamid

**Affiliations:** Institute for Public Health, National Institutes of Health, Ministry of Health, Blok B5 & B6,Kompleks NIH, No. 1, Jalan Setia Murni U13/52, Seksyen U13 Setia Alam, Shah Alam, 40170 Selangor Malaysia

**Keywords:** Malaysia; National Health and Morbidity Survey (NHMS) 2023, Non-communicable diseases, Methodology, Epidemiology, Epidemiology, Population screening

## Abstract

The National Health and Morbidity Survey (NHMS), which focuses on non-communicable diseases (NCDs), their risk factors and healthcare demand, is a cross-sectional, nationwide, population-based survey conducted every four-year cycle since 2011. NHMS 2023 was the 7th cycle of NCDs, their risk factors and healthcare demand, which aimed to monitor trends in diseases and healthcare utilisation. This article outlines the methodology, sociodemographic characteristics, and overall findings from the population surveyed in 2023 regarding NCDs, their risk factors, and various health-related topics. This survey utilised a two-stage stratified random sampling design and encompassed fifteen main scopes, with sample sizes calculated to meet the specific requirements of each scope. Conducted from July to September 2023, it involved the random selection of 499 enumeration blocks(EBs) by the Department of Statistics Malaysia (DOSM) across all states and federal territories in Malaysia. The step resulted in the selection of 5,988 living quarters (LQs). Data collection methods included validated questionnaires administered through face-to-face interviews and self-administered questionnaires, alongside clinical assessments and blood investigations using calibrated devices. To ensure the high quality of the collected data, we implemented stringent quality control measures. Furthermore, we applied complex sampling analyses to ensure that the findings accurately represented the population in Malaysia. The survey received responses from 13,616 individuals out of the 5,006 LQs that were successfully visited, resulting in an overall response rate of the survey of 83.2%, which includes individuals aged 5 years and above. A substantial segment of the population lived in urban regions, comprising 77.6% of the total. About half of the population consisted of males (51.9%), Malay (54.6%), and individuals who had attained secondary education (54.2%). Additionally, 20.0% of the population were within the age range of 30 to 39 years. The weighted prevalence of diabetes was 15.6%, hypertension 29.2%, hypercholesterolaemia 33.3%, overweight and obesity 54.4%. Depression among adults was 4.6%, while mental health problems among children were reported at 16.5%. The current tobacco smokers prevalence was 19.0%, and 29.9% of adults in Malaysia were physically inactive. The known asthma prevalence among adults was 6.2%, while the current asthma among children was 3.4%. Additionally, 37.7% of adults experienced sleep insufficiency. The results indicate that the higher proportion of diabetes, hypertension, and hypercholesterolaemia, particularly among the young age group, and the high prevalence of overweight and obesity, as well as mental health problems,  are making the healthcare system deal with more non-communicable diseases, complications, and problems. The methodology of this survey is robust for a population-based study. We took comprehensive steps to ensure the validity and reliability of the findings. The findings indicate an urgent need for targeted public health interventions to address the escalating health issues. By focusing on prevention and early intervention strategies, we can mitigate the impact of NCDs on the healthcare system and improve overall population health.

**Supplementary Information:**

The online version contains supplementary material available at 10.1038/s41598-025-08311-9.

## Introduction

Non-communicable diseases(NCDs) account for 75% of global mortality, making them the leading cause of death^1^. These present considerable challenges, especially in countries with developing economies. In Malaysia, non-communicable diseases account for approximately two-thirds of the total disease burden and premature mortality, which highlights their substantial effect on the nation’s health^2,3^. High-quality data from the National Health and Morbidity Survey (NHMS) is essential for effectively addressing the challenge posed by NCDs.

The Institute for Public Health (IKU), under the Ministry of Health Malaysia, initiated the NHMS in 1986. The objectives were to present the latest data regarding Malaysia’s health issues, disease burden, healthcare needs and health expenditure,. Initially conducted on a 10-year cycle, the Institute for Public Health has since 2011 implemented it annually in a 4-year cycle. The first year of each cycle focuses on NCDs, their risk factors, healthcare demand, and additional topics as requested by stakeholders, while the following years cover other key health areas set by the Ministry of Health. The NHMS 2023 represented the seventh cycle of the NHMS NCDs, their risk factors and healthcare demand^4^. Starting in 2023, the NHMS has moved from a 4-year cycle to a 5-year cycle.

The NHMS monitors disease trends and changing patterns of diseases and their related risk factors of the population in Malaysia. It also evaluates the effectiveness of health initiatives and provides data for decision-makers to develop strategies and allocate resources efficiently. In addition to this , NHMS contributes to the global evidence base by aligning with international NCD surveillance efforts, such as the WHO STEPwise approach^5^ and regional health surveys, making its findings comparable to those of other Southeast Asian (SEA)  countries such as Singapore and Indonesia^6,7^. The NHMS findings are crucial for monitoring national and global health indicators and assessing progress towards global commitments, including the Sustainable Development Goals (SDGs) and Universal Health Coverage (UHC)^3^.

Meanwhile, the National Strategic Plan for Non-communicable Diseases (NSP-NCD)  in Malaysia was developed in line with the WHO Global Action Plan for NCD Prevention and Control to strengthen Malaysia’s response to NCD prevention and control^8^. The NHMS 2023 identified five of the seven targets outlined in the NSP-NCD 2016–2025 that need to be assessed to determine whether Malaysia is on track to achieve them by 2025^8^. These targets include the prevalence of diabetes and obesity, elevated blood pressure, current tobacco use, harmful alcohol use, and insufficient physical activity^8^.

 The NHMS 2023  brings important updates, such as a more detailed lipid profile assessment to evaluate better heart health risks and the use of offline REDCap for safe, efficient and effective data collection. Notably, a new module on sleep insufficiency was incorporated to address a growing but underexplored public health issue in Malaysia. Emerging evidence indicates that inadequate sleep is increasingly being recognised as an independent risk factor for various NCDs, including cardiovascular disease, diabetes, and mental health disorders^9,10^. Despite its significance, national-level data on sleep insufficiency among adults in Malaysia is limited. The inclusion of this module aimed to fill this gap by providing robust, population-based evidence to inform future public health policies and interventions targeting sleep health. In addition, the asthma module was reintroduced after 17 years, offering fresh insights into asthma trends. 

 This article describes the methodology, sociodemographic characteristics, and key findings of the NHMS 2023 related to NCDs, their risk factors, and associated health issues in Malaysia. It aims to provide a peer-reviewed summary that supports scientific citation, promotes wider dissemination, and informs future research and policy development.

## Methods

### Study design, sampling frame and sampling design

**Fig. 1:**
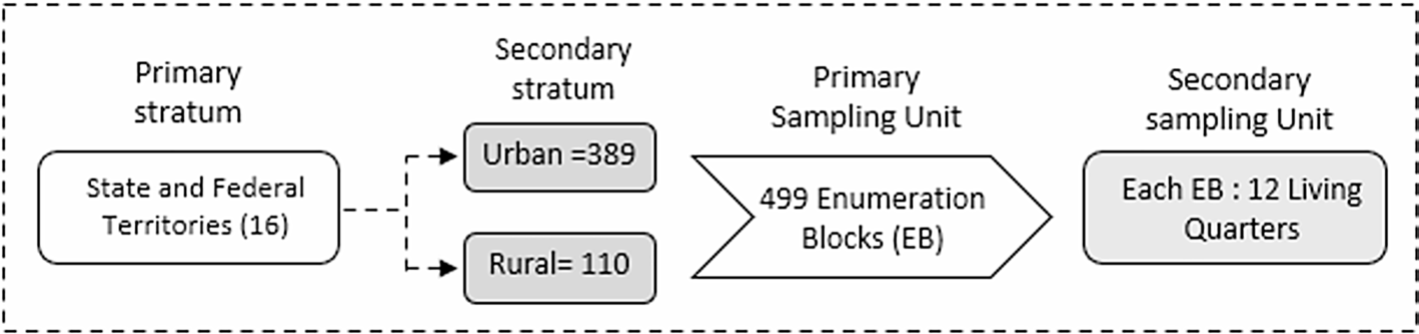
NHMS 2023 applied a two-stage stratified random sampling design.

This study was a cross-sectional, nationwide, population-based epidemiological survey conducted in Malaysia. The sampling frame was obtained from the 2020 National Population and Housing Census to ensure up-to-date population representativeness. A total of 5,988 living quarters were chosen at random by the Department of Statistics Malaysia (DOSM) from 499 selected enumeration blocks (EBs) throughout all states, including federal territories in Malaysia. Malaysia’s geographical regions have been divided into EBs, each typically comprising 80–120 Living Quarters (LQs) and containing an average population of 500–600 individuals^22^. EBs were categorised as urban areas if the regions, including their surrounding areas, had a population of 10,000 or more and extended to special development areas, which are distinct from officially gazetted or built-up regions larger than 5 km², as defined by DOSM^23^ These areas must have a minimum population of 10,000 residents, with at least 60% of individuals aged 15 years and above engaged in non-agricultural activities^23^. Conversely, rural areas are identified as geographical regions with a population of less than 10,000. To ensure its national representativeness, this survey employed a two-stage stratified random sampling design. The primary stratum consists of all the states in Malaysia, including the federal territories, as shown in Fig. 1, while the secondary stratum consists of urban and rural areas within the primary stratum.  In the first stage, 499 EBs, comprising 389 urban and 110 rural areas, were randomly selected by the Department of Statistics Malaysia (DOSM). In the second stage, all eligible living quarters within each selected EB were included as secondary sampling units. The target population included all non-institutionalised residents of Malaysia aged five years and above who had resided in the selected living quarters for at least two weeks prior to the survey and were able to communicate independently or through a proxy. The sample was designed to yield approximately 20,000 respondents.

### Scope selection process

 Initially,  all the relevant divisions under Ministry of Health’s stakeholders received a formal request letter from IKU for new scope suggestions prior to the implementation of NHMS.  The IKU then received the suggested scopes for selection. The research team conducted a thorough literature search and prepared evidence tables to identify, justify, and select the relevant scope for the survey. The research team used the following criteria to select the topics^11^:

- The issue/problem is either currently widespread or has the potential to become highly prevalent.
- The issue/problem is linked to factors such as affluence, lifestyle, environmental conditions, and demographic shifts.
- The issue/problem leads to considerable physical, mental, or social disabilities.
- The issue/problem has significant economic impacts.
- The necessary information cannot be obtained through routine monitoring systems.
- Gathering this information is best accomplished through a community survey, and it is feasible to do so. 

We conducted several engagement sessions with stakeholders to discuss, finalise, and refine the scope. An example of the evidence table used for scope selection is provided in Supplementary Table S1. The final proposed scope was presented to the steering committee, chaired by the Director General of Health, for approval as shown in Table 1.

**Table 1:** Scopes, instruments, methods of data collection, devices, target age groups, and level of analysis, NHMS 2023.

No.	Scopes	Instruments	Method	Target age group	Devices	Level of analysis
1	Sociodemography	Derived from NHMS 2019	Face-to-face	All respondents	–	State
2	Diabetes	STEPS^5^	Face-to-face	≥ 18 years	AccuChek Guide (Capillary Blood Glucose)	State
3	Hypertension	STEPS^5^	Face-to-face	≥ 18 years	OMRON 907	State
4	Hypercholesterolaemia	STEPS^5^	Face-to-face	≥ 18 years	Cardiochek PA (Capillary Blood Cholesterol)	State
5	Physical Activity	GPAQ^12^ considered 3 domains; work, leisure, and travel	Face-to-face	≥ 18 years		State
6	Smoking and Tobacco Use	Modified version of GATS	Face-to-face	≥ 15 years		State
7	Health Screening	–	Face-to-face	≥ 18 years		State
8	Dietary Practices	STEPS^5^	Face-to-face	≥ 18 years		State
9	Disability	WG Short Set^13^—having “some difficulty” in two domains or “a lot of difficulty” or “cannot do at all” in one domain	Face-to-face	≥ 18 years		State
10	Asthma (Adults)	ECRHS ^14^	Face-to-face	≥ 18 years		National (urban and rural)
11	Asthma (Children)	ISAAC ^15^	Face-to-face	6–17		National (urban and rural)
12	Nutritional status (Anthropometric)	WHO 1998^16^ and CPG 2023^17^ cut-offs		≥ 18 years	SECA Electronic Weighing Scale (SECA 813) SECA Portable Stadiometer (SECA 213) SECA measuring Tape 201	State
		WHO Growth Reference 2007^38 ^		15–17 years	SECA Electronic Weighing Scale (SECA 813) SECA Portable Stadiometer (SECA 213)	National
13	Mental Health (Adults)	PHQ-9 ^18^ Depressive symptoms: Total score of PHQ-9 ≥ 10	Self-administered	≥ 16 years		National (urban and rural)
14	Mental Health (Children)	SDQ^19^ Total difficulty ≥ 14	Self-administered	5–15 years		National (urban and rural)
15	Alcohol	AUDIT^20^ Low-risk drinkers: 0–7 Risky drinkers: 8–19 Probable alcohol drinkers: ≥20	Self-administered	≥ 13 years		National (urban and rural)
16	Insufficient sleep	BRFSS ^21^ < 7 h	Face-to-face	≥ 18 years		State

*STEPS* The WHO STEPwise approach to NCD risk factor surveillance, *GPAQ* Global Physical Activity Questionnaire, *GATS* The Global Adult Tobacco Survey, *WG Short Set* Washington Group Short Set, *ECRHS* European Community Respiratory Health Survey, *ISAAC* International Study of Asthma and Allergies in Childhood, *PHQ-9* Patient Health Questionnaire-9, *SDQ* Strengths and Difficulties Questionnaire, *BRFSS* Behavioural Risk Factor Surveillance System, *AUDIT* Alcohol Use Disorders Identification Test.

### Sample size calculation

We calculated the sample size to meet the requirements of each of the fifteen scopes , with sample sizes tailored accordingly (Table 2). A single proportion formula n _srs_ ≥(Z² *_α/2_ *P(1 – P)) / e² for estimating prevalence was used to determine the sample size, based on published prevalence, with a margin of error of 0.01 to 0.07 and a confidence interval of 95%. The size was adjusted to account for design effect (2.0) and non-response (35%), then further adjusted for national or state-level analysis. The design effect for each scope was estimated using the previous NHMS. A design effect ranging from 1 to 3 is generally indicative of a well-designed study^24^.

**Table 2:** Required sample size by scope of the study for different precisions.

Scope	Prevalence (%) (NHMS 2019 or other literature)	Minimum sample size per strata	Number of strata	Total required sample size
Diabetes (undiagnosed)	8.9	455	16	7280
Hypertension (undiagnosed)	14.1	372	16	5952
Hypercholesterolaemia (known)	13.5	359	16	5744
Physical activity	25.1	401	16	6416
Smoking and tobacco use	21.3	426	16	6816
Alcohol	11.1	358	2	716
Health screening-medical check-up	49	392	16	6272
Adequate fruit intake	9.4	430	16	6880
Adequate vegetable intake	10	432	16	6912
Inadequate water intake	49.4	392	16	6272
Disability	11.1	374	16	5984
Asthma (adults)	6.4	681	2	1362
Asthma (children)	7.1	582	2	1164
Nutritional status (adults)	19.7	449	16	7184
Nutritional status (15–17 y-o)	14.8	269	1	269
Mental health (adults)	2.3	1726	2	3452
Mental health (children)	7.9	546	2	1092
Insufficient sleep	35.5	359	16	5744

### Inclusion-exclusion criteria

The eligibility criteria included those aged 5 years old and above who resided in the non-institutional LQs for at least 2 weeks prior to the data collection. Excluded were people who lived in institutional settings like hotels, hostels, hospitals, etc. Respondents were also excluded if they had cognitive impairment and were unable to provide consent or respond to the interview, had language barriers that prevented communication, or were mute and/or deaf without a proxy or interpreter.

### Questionnaires, tools and quality control

Data collection was conducted from July to September 2023 through face-to-face interviews, self-administered questionnaires (SAQs), clinical assessments, and blood investigations using calibrated point-of-care testing devices. The validated questionnaires were adopted based on international standards, including the WHO STEPwise Approach to NCD Risk Factor Surveillance (STEPS), the Global Physical Activity Questionnaire (GPAQ), and the Patient Health Questionnaire-9 (PHQ-9) for mental health assessment, as shown in Table 1. Pre-testing, including cognitive debriefing, was conducted in January 2023 to assess the validity and reliability of the additional questionnaires. Pre-test training was provided to related key persons, and 30 respondents per module participated in the pre-test. The study team reviewed respondent feedback and revised the questionnaire wording where necessary.

Using REDCap mobile apps on tablets, the Computer-Assisted Personal Interview (CAPI) method was used to conduct the face-to-face interviews in either Malay or English. Data collectors administered the validated questionnaires through REDCap mobile apps during the interviews. Hard copies of sensitive questionnaires, specifically the Alcohol Use Disorders Identification Test (AUDIT) and the Patient Health Questionnaire (PHQ-9), were distributed to ensure respondent privacy and confidentiality. Respondents were informed that data collectors would collect the sealed envelopes with the completed surveys later that day or the following day. The same respondent ID used in the interview module was then used to enter data from the SAQs into the REDCap app.

Nurses or assistant medical officers took two readings of the anthropometric measurements, including height, weight, and waist circumference. The average of the two readings was used for the analysis.  Height was measured using the SECA Portable Stadiometer 213 in a standing position; weight was measured using the SECA Electronic Weighing Scale (SECA 813), and waist circumference was measured using the SECA measuring Tape 201. The Accu-Chek Guide device was used to measure capillary glucose, while the CardioChek^®^PA device was used for capillary cholesterol. In addition, this was the first NHMS cycle to include an expanded lipid profile assessment during the survey. All devices and test strips were stored in a sealed cooler bag to maintain the recommended temperature, prevent overheating and ensure optimal performance.

The Omron Digital Automated Blood Pressure Monitor Model HEM-907 was used for blood pressure measurement. Respondents were instructed to rest for 15 minutes prior to measurement, refrain from physical activity, and avoid stimulants such as caffeine and smoking. Blood pressure was measured three times, with a three-minute interval between readings, while the respondent was seated calmly, with the right arm positioned at heart level^5^.

Quality control measures were strictly enforced, including data collectors’ training, instrument calibration, and real-time data monitoring. To ensure consistent, standardised and effective data collection, field supervisors, team leaders, nurses or assistant medical officers, and data collectors completed a comprehensive seven-day training programme. Initial in-house training for field supervisors was held in June 2023 at IKU, followed by regional training sessions conducted from 4 to 10 July 2023 in Sabah and Sarawak and 12 to 18 July 2023 in Peninsular Malaysia. The training covered survey content, procedures, and team responsibilities and included briefings, simulated interviews, and supervised practice sessions. Nurses and assistant medical officers also received specific instruction on clinical assessments, device handling, quality checks of devices and referral guidelines. Data collectors underwent comprehensive REDCap training to ensure accurate entry of each respondent’s identification number and other data into the REDCap apps. Upon completion of the training, a pilot data collection activity was conducted to test field procedures and identify any issues, which were then addressed to ensure smooth implementation during the actual data collection phase.

To ensure that all required questions and responses were properly captured, the questionnaire’s skip patterns and the flow of the programme were integrated into the tablet. The built-in quality control system ensured that all responses were recorded accurately before completing the interview by allowing only valid inputs based on predefined answer options and acceptable ranges for continuous variables. All machines were calibrated before usage, and quality control procedures for the Accu-Chek Guide and CardioChek^®^PA were performed weekly using specific control solutions by the nurses or assistant medical officers. These procedures were properly documented using designated forms. Field supervisors conducted verification interviews with 5% of respondents to ensure data accuracy, consistent with international practices, where 5%^25^ –10%^26^ of households are typically rechecked as part of standard quality assurance protocols.

Field supervisors also reviewed the quality of each record to ensure accuracy before submitting data to the server via the REDCap system. The task included verifying critical variables such as respondent IDs from the household and field retention forms, as well as clinical measurement variables, which were cross-checked against entries in the clinical assessment book.

Despite rigorous planning, several logistical, operational, and environmental challenges may have introduced potential biases in the NHMS 2023 data collection process. Difficult terrain and transportation issues posed accessibility challenges, particularly in rural and remote areas.In urban settings, gated communities also hindered access to respondents due to restricted entry. Additionally, safety concerns, such as unsafe zones (“black areas”), aggressive animals, and unpredictable weather conditions like heavy rainfall and flooding, further complicated field operations. Respondent-related issues, including lack of cooperation, suspicions of scams, aggressive behaviour, and language barriers, also contributed to potential response biases.

Data was transmitted to the National Institutes of Health central server via CAPI using the REDCap system. A dedicated data management team consistently assessed the data completeness and quality. The team performed data cleaning upon receipt before forwarding the dataset to the analysis team. Weekly data downloads from the server supported quality control efforts by verifying respondent IDs, identifying outliers, and detecting erroneous entries. Any outliers and discrepancies were identified and verified with field teams to ensure data accuracy. The Central Coordinating Team (CCT), led by the Director of the Institute for Public Health, conducted weekly progress reviews during regular meetings. Field operations were monitored by comparing the targeted number of LQs with weekly progress reports, the volume of data uploaded to the server, and any reported logistical issues. An online field monitoring board using Google Sheets was used to track weekly progress of data collections teams throughout the data collection phase.

### Field implementation of the survey

Data collectors conducted the interview sessions at the respondent’s house using mobile applications on tablet during the data collection phase. Field supervisors closely monitored and supervised these sessions.. Each respondent received an information sheet and consent form before the start of the interview. The survey’s purpose, process, and requirements were explained to each respondent.

Locked LQs, unoccupied spaces, refusals to participate, and hostile or unsafe environments were considered non-response household survey situations. Respondents who were absent during data collection, refused participation, or faced language barriers were considered unsuccessful cases.

Nurses or assistant medical officers properly documented the clinical measurements and anthropometric measurements in a clinical assessment book and simultaneously entered the data into the REDCap system. Individuals who screened positive for elevated blood glucose, high blood pressure, high cholesterol, or uncontrolled diabetes or hypertension were issued referral letters and advised to seek further assessment and follow-up care at the nearest government health facility.

To improve logistics and operational efficiency, the 36 data collection teams were divided into seven zones based on geographical distribution across states, with 27 teams in Peninsular Malaysia and 9 teams in Sabah, Sarawak and Labuan. Each team consisted of a team leader, another research assistant, a driver, and either an assistant medical officer or a nurse.

Team allocation to each zone was determined based on the number of selected EBs and LQs provided by DOSM. NHMS 2023 received strong support from the respective state health departments in preparing for field operations. A liaison officer was appointed in each state from the state health department to facilitate data collection operations. Detailed methodology and data collection have been described in the NHMS 2023 Technical Report​^4^.

### Ethical approval and consent to participate

All respondents provided written informed consent before taking part in the interviews. To ensure confidentiality, all data were anonymised prior to analysis. For illiterate respondents, consent was obtained from their legal guardians or representatives, with thumbprint impressions provided in the presence of a literate witness. The study protocol received ethical approval from the Medical Research and Ethics Committee (MREC), Ministry of Health Malaysia (22-00545-XAC(2)). All methods adhered to the principles of the Declaration of Helsinki and the Malaysian Good Clinical Practice Guidelines.

### Statistical analysis

Data analysis was conducted using IBM’s Statistical Package for Social Sciences (SPSS) software for Windows, version 29.0 (IBM Corporation, Armonk, NY, USA). Before data analysis, data verification and cleaning processes were performed. We used complex sampling analyses to incorporate sampling weights in all analyses, ensuring accurate representation of the general population in the data. The weights were calculated to address variations in selection probability, non-response, and the main sociodemographic characteristics.

The sample weight calculation for the survey included three main steps: (1) calculation of the design weight, calculated from all steps of random selection by each stratum in the design; (2) an adjustment of non-response by LQs and sample individuals in the survey; and (3) post-stratification calibration adjustment of sample weights to correct for deficiencies in how the survey sample estimates the population. Post-stratification adjustments were conducted according to age, sex and ethnicity, using 2023 population estimates from the DOSM. These steps ensured that the survey findings were representative of Malaysia’s current population.

## Results

**Fig. 2:**
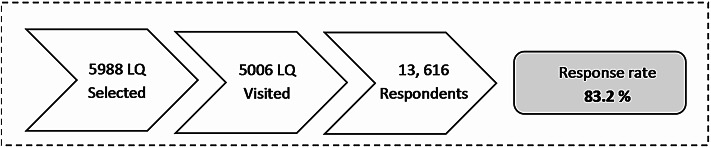
Response rates by living quarters and individuals by states, NHMS 2023.

An overall response rate of 83.2% was achieved. A total of 13,616 respondents aged 5 years and above were successfully interviewed (individual response rate: 93.2%), from 5,006 successfully visited LQs (LQ response rate: 90.1%), as shown in Fig. 2.

We distributed the sample proportionately to the population size in all Malaysian states. Table 3 presents the characteristics of the study population. The states with bigger populations had a bigger sample, while the states with smaller populations had a smaller sample. Selangor (22.5%) had the biggest sample, followed by Johor (12.2%) and Sabah (10.8%); meanwhile, Labuan (0.3%) had the smallest sample. The total number of respondents was 13,616 , of whom 51.9% were male and 48.1% were female. More than half (54.6%) were Malay, followed by Chinese (20.5%), ‘Others’ ethnicity (8.5%), Bumiputera Sabah (6.7%), Indian (5.6%) and Bumiputera Sarawak (4.1%). Marital status was as follows: married/living with a partner (49.1%), never married (44.2%), and separated/divorced/widowed (6.7%). For educational levels, more than half (54.2%) had completed secondary school, 20.7% had no formal education, 13.5% had completed primary school, and only 11.6% had tertiary education. Household income was calculated based on reported individual incomes and subsequently classified according to state-specific thresholds for the Bottom 40 per cent (B40), Middle 40 per cent (M40), and Top 20 per cent (T20) household groups in Malaysia, as defined by DOSM^27^. The findings revealed that 60.4% were classified inside the B40 family income category, while merely 12.5% fell into the T20 category. This low percentage of the T20 household income category could be attributed to the underreporting of the self-reported income, as experienced by any other survey in other countries^28^.

**Table 3:** Sociodemographic characteristics of the population, NHMS 2023.

Sociodemographic characteristics	Count	Weighted percentage (%)
Malaysia	13,616	100.0
**State**		
Johor	1282	12.2
Kedah	662	6.3
Kelantan	752	5.5
Melaka	748	3.1
Negeri Sembilan	744	3.6
Pahang	720	4.6
Pulau Pinang	644	5.4
Perak	684	6.9
Perlis	643	0.9
Selangor	1726	22.5
Terengganu	687	3.6
Sabah	975	10.8
Sarawak	956	7.7
WP Kuala Lumpur	691	6.3
WP Labuan	942	0.3
WP Putrajaya	760	0.4
**Location**		
Urban	10,402	77.6
Rural	3214	22.4
**Sex**		
Male	6426	51.9
Female	7190	48.1
**Age group (years**)		
5–9	1185	8.1
10–19	1903	17.4
20–29	1780	17.3
30–39	2056	20.0
40–49	1907	12.2
50–59	1806	12.7
60 and above	2979	12.3
**Ethnicity**		
Malay (included Orang Asli)	8217	54.6
Chinese	1909	20.5
Indian	839	5.6
Bumiputera Sabah	1366	6.7
Bumiputera Sarawak	512	4.1
Others	773	8.5
**Marital status**		
Never married	5004	44.2
Married/living with a partner	7252	49.1
Separated/divorced/widowed	1351	6.7
**Educational level**		
No formal education	2955	20.7
Primary education	1985	13.5
Secondary education	7267	54.2
Tertiary education	1373	11.6
**Occupation**		
Government employee	994	5.6
Private employee	3205	29.1
Self-employed	1651	12.7
Unpaid worker/Homemaker/caregiver	2032	13.0
Retiree	836	4.2
Student	2735	23.2
Not working included those were unemployed, old age, not working due to health problems, and children who did not attend school	2115	12.2
**Household income category**		
B40	8528	60.4
M40	3466	27.0
T20	1585	12.5

**Fig. 3:**
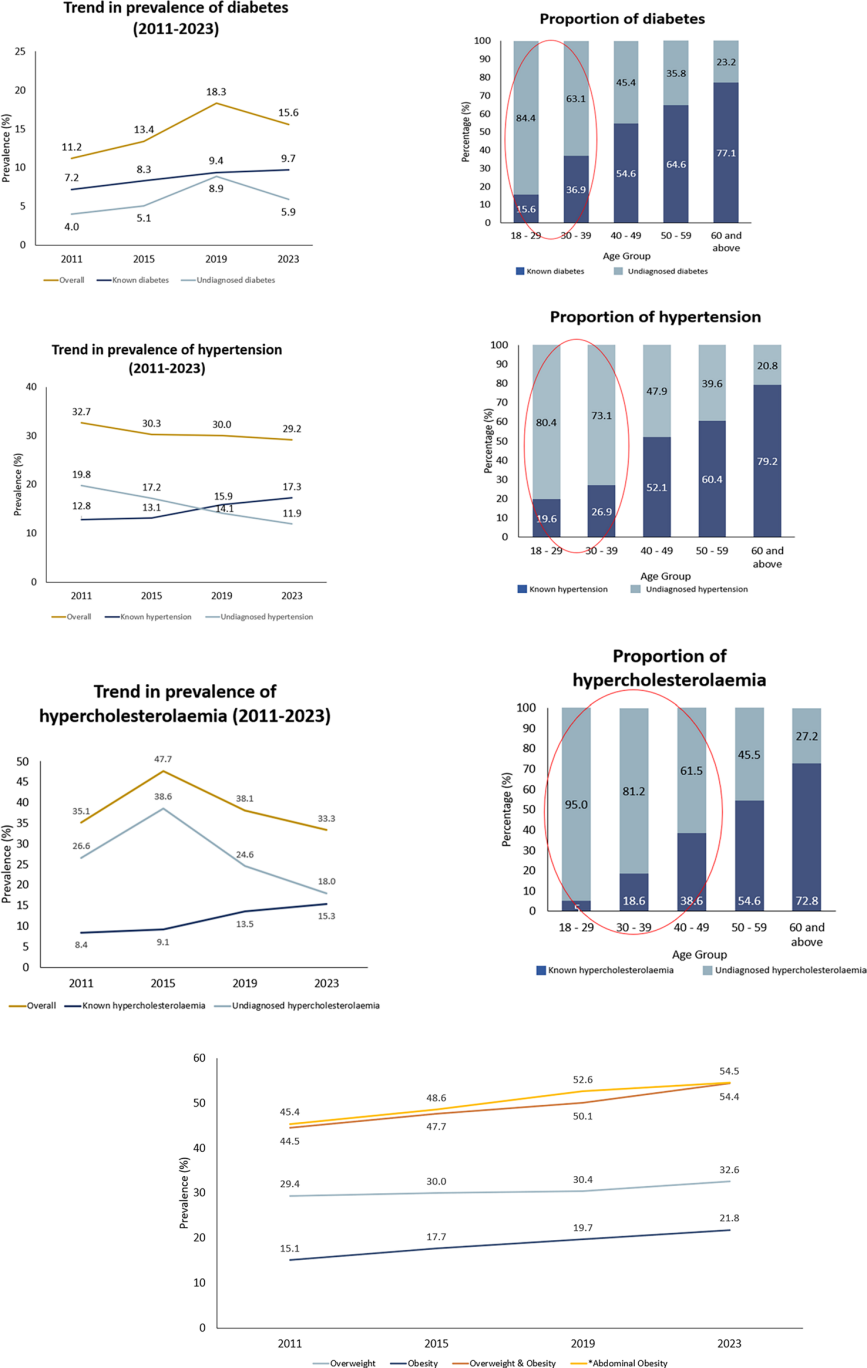
Trends in diabetes, hypertension, hypercholesterolaemia,  overweight and obesity from 2011 to 2023.

The NHMS 2023 findings provide an evolving overview of non-communicable diseases (NCDs) in Malaysia, as presented in Table 4. Key findings include a diabetes prevalence of 15.6%, hypertension 29.2%, hypercholesterolaemia 33.3%, obesity 21.8%, physical inactivity 29.9%,current tobacco smokers 19.0%, and depression among adults 4.6%. Diabetes prevalence reached 15.6% in 2023, continuing an upward trend from 11.2% in 2011 and peaking at 18.3% in 2019^4^, as shown in Fig. 3. Hypertension prevalence remained stable at 29.2%, showing a plateau since 2011 (Fig. 3). In contrast, hypercholesterolaemia declined slightly to 33.3% from 35.1% in 2011^4^ (Fig. 3). The younger demographic showed a higher proportion of undiagnosed diabetes, hypertension, and hypercholesterolaemia^4^ (Fig. 3).

**Table 4:** Main NCDs and their risk factors findings from the NHMS 2023.

Health-related findings	Weighted prevalence (%)
**Diabetes**	
Overall	15.6
Known diabetes	9.7
Raised blood glucose among those not known to have diabetes	5.9
**Hypertension**	
Overall	29.2
Known hypertension	17.3
Raised blood glucose among those not known to have hypertension	11.9
**Hypercholesterolaemia**	
Overall	33.3
Known hypercholesterolaemia	15.2
Raised blood glucose among those not known to have hypercholesterolaemia	18.1
Overweight	32.6
Obesity	21.8
Abdominal obesity	54.5
**Physical activity**	
Physically inactive	29.9
Sedentary behaviour	49.9
**Dietary practices**	
Inadequate fruit/vegetable intake of 5 servings per day	95.1
Inadequate water intake of 6 glasses per day	22.2
**Smoking and tobacco use**	
Current tobacco smokers	19.0
Current e-cigarette users	5.0
**Alcohol consumption**	
Current alcohol drinkers	10.4
The proportion of binge drinkers among current drinkers*	45.1
**Mental Health**	
Depression among adults	4.6
Mental health problems among children aged 5 to 15 years old	16.5
**Health screening**	
Mammogram examination (past 3 years)	12.8
Pap smear examination (past 3 years)	34.9
NCDs health screening/medical check-up (past 1 year)	57.2
Faecal occult blood test	6.8
**Asthma**	
Known asthma among adults	6.2
Probable asthma among adults	2.7
Current asthma among children (6–17 years)	3.4
**Disability**	
Overall disability among adults	8.2
Overall difficulty among adults	21.7
Insufficient sleep	37.7

*percentage.

Obesity and overweight continue to rise, now affecting 54.4% of adults, marking a 22% increase since 2011^4^ (Fig. 3). Abdominal obesity shows a similar trend,reaching 54.5%. A graphical representation of trends for diabetes, hypertension, hypercholesterolaemia, and overweight and obesity, from 2011 to 2023 is presented in Fig. 3 to illustrate these patterns over time. While physical inactivity has decreased to 29.9%, sedentary behaviour remains high at 49.9%^4^. Worryingly, only 4.9% of adults consume adequate fruits and vegetables. Although the prevalence of current tobacco smokers has declined to 19.0%, it remains concerning, along with a 5.0% prevalence of e-cigarette use^4^.

Mental health issues are on the rise, with depression prevalence among adults doubling since 2019 to 4.6% and mental health problems among children reaching 16.5%. Since 2006, known asthma prevalence among adults has increased to 6.2%, whereas the prevalence of current asthma among children has decreased to 3.4%. Despite a decline in disability prevalence (8.2%), utilisation of rehabilitation service remains low^4^. Additionally, sleep insufficiency was reported by 37.7% of adults.

## Discussion

The NHMS 2023 was a nationwide survey focused on NCDs, aimed at providing population-level insights into priority areas identified by the Ministry of Health. A response rate of 83.2% reflects a robust and effective survey design, meticulous fieldwork, and the trust of the respondents in the survey. This rate is higher than that of many other conducted population-based surveys, which typically report response rates between 60 and 80%, depending on the type of survey and survey methodology^29,30^. Several factors likely contributed to the high response rate, including well-trained data collectors, comprehensive community engagement, and the use of face-to-face interviews. These elements enhanced data representativeness, reduced the non-response bias, and ensured more accurate reflectionsof the population’s health status in Malaysia. Good collaboration and support from the state health departments and district health offices also played a key role in facilitating a favourable response rate during the fieldwork. However, despite the high overall response rate, non-response may still occur among specific target groups, particularly those residing in urban areas and higher-income households.

The NHMS 2023 survey utilised validated questionnaires, including the WHO STEPwise Approach to Surveillance (STEPS), the Global Physical Activity Questionnaire (GPAQ), and others, as shown in Table 1^5^. These instruments are widely recognised and have been applied in national surveys conducted in countries such as Singapore, Brunei, and Indonesia^31,32^. This approach ensures standardisation, enabling the findings from the NHMS 2023 to be comparable with those from other countries globally.

The findings reveal a high prevalence of diabetes, hypertension, hypercholesterolaemia, overweight and obesity, and mental health problems, with particularly higher proportion of undiagnosed diabetes, hypertension, hypercholesterolaemia among younger populations. These undiagnosed cases pose significant risks, including early complications, increased cardiovascular disease burden, reduced productivity, and financial strain on healthcare systems. When compared to regional peers,  the findings align with trends observed in other SEA countries. For instance, Singapore’s National Population Health Survey 2022 reported similar patterns of undiagnosed cases of diabetes, hypertension and hyperlipidaemia, particularly among younger age groups^33^. These comparisons highlight shared public health challenges in the region and emphasise the value of NHMS 2023 data for regional benchmarking and cross-country learning. The prevalence of overweight and obesity has increased by 22% since 2011, now affecting more than half the adult population. This trend likely contributes to increasing burden of NCDs such as type 2 diabetes and stroke. Malaysia’s prevalence of overweight and obesity are higher than those of most other SEA countries^37^. Mental health issues have also doubled, with depression now affecting 4.6% of adults and mental health problems impacting 16.5% of children compared to 2019. The latest NHMS 2023 data shows that known asthma prevalence among adults in Malaysia has risen from 4.5% in 2006 to 6.2% in 2023, with an additional 2.7% considered probable asthma, totalling 8.9%​^4^. This reflects the current national burden of asthma. Awareness is generally higher in urban areas, likely due to better access to healthcare. These findings indicate that greater efforts are needed to reduce risk factors, including secondhand smoke exposure and obesity, while also enhancing public awareness and ensuring accessible diagnosis and treatment, particularly in rural and underserved communities^4^. In addition, sleep insufficiency, experienced by 37.7% of adults in Malaysia, has emerged as a significant public health concern, as inadequate sleep is increasingly linked to obesity, cardiovascular disease, diabetes, mental health disorders, and accidents^9,10,34^.

A whole-of-government approach is essential in addressing these challenges. Initiatives like the Agenda Nasional Malaysia Sihat (ANMS) play a crucial role in promoting healthy lifestyles and supporting early disease detection, as introduced by the Ministry of Health Malaysia. In 2023, as part of ANMS, the National Health Screening Initiative (NHSI) expanded its screening programmes to include individuals aged 18 and above^35^. Expanding the age threshold across all screening programmes would strengthen early detection efforts, enabling timely intervention and more effective disease management. This approach may also help reduce the number of undiagnosed cases, especially among younger populations, where higher proportions of undiagnosed cases have been observed. Early identification leads to better health outcomes and lower long-term healthcare costs, supporting a proactive strategy in tackling NCDs in Malaysia. Multi-sectoral collaboration focuses on promoting physical activity, encouraging healthier environments and lifestyles, and ensuring food security to address issues such as obesity and mental health. Effort to strengthen health literacy, adopt digital technologies, and foster cross-sector collaboration, particularly with the education sector, are also important to reducing the burden of NCDs in Malaysia.

The findings should be interpreted with consideration of certain limitations, such as adjustments for weighting and the under-representation of certain ethnic groups. As a cross-sectional study, it cannot determine causation for the observed outcomes. Additionally, relying on self-reported responses introduces potential recall bias and lacks external validation mechanisms^36^. Furthermore, the survey results may have been impacted by response biases that occurred during the data collection process, as covered in the questionnaire, tools and data quality section. We enforced strict quality control measures to reduce these data collection biases. Routine calibration managed device malfunctions, and strategic scheduling assisted in navigating adverse weather conditions. Additionally, proactive community engagement- including negotiations with Joint Management Bodies(JMBs) or resident associations, strong collaboration with the police, state health departments and district health offices, as well as public awareness efforts- improved response rates and ensured a more representative data. Trained data collectors ensured respondents understood the questions without altering the validated questionnaire content. Publicity efforts, including the official launch by the former Minister of Health, media coverage, and social media promotion, likely contributed to greater public awareness and a higher response rate.

The use of offline REDCap apps made the data collection process smoother and more reliable, ensuring accurate and secure data even in remote areas with poor internet access. Its easy-to-use design helped field teams manage data efficiently, with real-time checks reducing mistakes during entry. The ability to work offline meant there were no disruptions, while automatic backups kept data safe. With proper training, field teams could handle data seamlessly, making REDCap a practical and trustworthy tool for managing large-scale surveys like NHMS 2023.

Regardless of these challenges, the survey remains a key resource as the latest national representative study offering baseline information on health issues related to non-communicable diseases. Many channels, including mass media, have been used to disseminate the findings.

## Conclusions

This survey’s methodology is robust for a population-based study. Comprehensive steps ensured the findings were valid and reliable. These results will help the Ministry of Health to review programmes, develop policies, and plan resources to improve health outcomes in Malaysia. The findings indicate an urgent need for targeted public health interventions to address the escalating health issues. By focusing on prevention and early intervention strategies, we can mitigate the impact of non-communicable diseases on the healthcare system and improve overall population health.

## Electronic supplementary material

Below is the link to the electronic supplementary material.

Supplementary Material 1

## Data Availability

For data protection purposes, the data used for this study are not publicly available due to local ethics regulations and could be obtained via written permissions to the Director General of Health, Malaysia. The corresponding author can be contacted for further information to retrieve the dataset analysed in this study.
