# Supplementary material for: Conducting the National Health and Morbidity Survey 2023 in Malaysia with focus on methodology and main findings on non-communicable diseases
Source: Sci Rep. 2025 Jul 15;15:25531. doi: 10.1038/s41598-025-08311-9 (PMC12264187; doi:10.1038/s41598-025-08311-9)
Supplement: Supplementary file 1 — Supplementary Material 1 [file 41598_2025_8311_MOESM1_ESM.docx]

| **#** | **Scope** | **Target Population** | **Justification** | **Literature review** | | **Methodology** | **Decision**  **(Accept/**  **Reject)**  **Reject** |
| --- | --- | --- | --- | --- | --- | --- | --- |
|  |  |  |  | **Indicator/ Variables** | **Prevalence** | **Tools** |  |
| 1 | Self-Monitoring Devices- to be integrated in the existing module  - Self-Monitoring Blood Sugar- in a diabetes module  - Home Blood Pressure Monitoring- in a hypertension module  ( HBPM ) | 18 years and above | - To determine the importance of using monitoring devices for self-monitoring among households at home. - To determine the frequency of monitoring device usage in a month. | - To identify whether the respondent has monitoring device at home - To determine the frequency of monitoring device usage | - Records of SBMG can be used during consultation with health care providers to titrate diabetic medications and to guide physical activity and food intake. The effectiveness of SBMG has been established for insulin-treated patients. Kirk, J. K., & Stegner, J. (2010). Self-monitoring of blood glucose: practical aspects. Journal of diabetes science and technology, 4(2), 435–439. <https://doi.org/10.1177/193229681000400225> - There is growing evidence that SMBP with guided support improves BP. Tucker, K. L., Sheppard, J. P., Stevens, R., Bosworth, H. B., Bove, A., Bray, E. P., Earle, K., George, J., Godwin, M., Green, B. B., Hebert, P., Hobbs, F., Kantola, I., Kerry, S. M., Leiva, A., Magid, D. J., Mant, J., Margolis, K. L., McKinstry, B., McLaughlin, M. A., … McManus, R. J. (2017). Self-monitoring of blood pressure in hypertension: A systematic review and individual patient data meta-analysis. PLoS medicine, 14(9), e1002389. <https://doi.org/10.1371/journal.pmed.1002389> | 1. Adakah anda mempunyai alat pemantauan berikut di rumah?  (Do you own the following monitoring devices at home?)  2. Jika Ya, berapa kerap anda menggunakan alat tersebut? (if yes, how often do you use the machine? Jawab SATU pilihan sahaja Answer ONE option only  __hari dalam seminggu/ days in a week  __ hari dalam sebulan/ days in a month  __tidak pernah  Pada hari anda menggunakan alat menggunakan alat periksa gula/darah, secara purata berapa kali dalam sehari anda menggunakannya? On the days that you use the blood sugar/ pressure machine, on average how many times in a day do you use it?  __kali dalam sehari/ times in a day | Accept |
| 2 | Asthma Among Adult and Child Populations | 18 years and above | - To determine the latest prevalence of asthma among adults and children in Malaysia, since the national data was in 2006 | Prevalence of asthma among adults and children in Malaysia | - The National Health and Morbidity Survey (NHMS) 2006 reported an asthma prevalence of 7.1% in the Malaysian population at that time. - In the United States National Health Survey, a four-item questionnaire is used to assess the prevalence of asthma. - For NHMS 2006, the International Study of Asthma and Allergies in Childhood (ISAAC) questionnaire, consisting of 21 items, was utilised to assess asthma prevalence specifically among the paediatric population. - For the adult population, the European Community Respiratory Health Survey (ECRHS) questionnaire was adopted. | -Check treatment card | Accept |
| 3 | Sleep component | 18 years and above | More evidence is coming on the relationship between mental health with CVD risk | Prevalence of sleep deprivation | - A total of 6,212 working adults (54.7%) reported getting less than 7 hours of sleep in Malaysia - Participants self-reported their sleep duration based on the single-item question “On average, how many hours of sleep do you get in 24 hours?” Insufficient sleep was defined as rest/sleep of <7 h per night on average over the past 30 days. Mean daily sleep time (in hours) for adult individuals was reported and categorised as inadequate sleep (<7 h of sleep) and adequate sleep (≥7 h of sleep). The categories were based on the American Academy of Sleep Medicine, the Sleep Research Society, and the National Sleep Foundation.   - Chan CMH, Siau CS, Wong JE, Wee LH, Jamil NA, Hoe VCW. Prevalence of Insufficient Sleep and Its Associated Factors Among Working Adults in Malaysia. Nat Sci Sleep. 2021;13:1109-1116. Published 2021 Jul 13. doi:10.2147/NSS.S295537 | Behavioural Risk Factor Surveillance System (BRFSS)  -Insufficient sleep was defined as res/sleep of < 7 hours per night on average over the past 30 days | Accept |
